# The Current Status of Usability Studies of Information Technologies in China: A Systematic Study

**DOI:** 10.1155/2014/568303

**Published:** 2014-06-19

**Authors:** Jianbo Lei, Lufei Xu, Qun Meng, Jiajie Zhang, Yang Gong

**Affiliations:** ^1^Center for Medical Informatics, Peking University, Haidian District, Beijing 100191, China; ^2^School of Biomedical Informatics, University of Texas Health Sciences Center at Houston, Houston, TX 77030, USA; ^3^Center for Statistics and Information, National Health and Family Planning Commission, Beijing 100810, China

## Abstract

*Objectives.* To systematically review and analyze the current status and characteristics of usability studies in China in the field of information technology in general and in the field of healthcare in particular.* Methods.* We performed a quantitative literature analysis in three major Chinese academic databases and one English language database using Chinese search terms equivalent to the concept of usability.* Results.* Six hundred forty-seven publications were selected for analysis. We found that in China the literature on usability in the field of information technology began in 1994 and increased thereafter. The usability definitions from ISO 9241-11:1998 and Nielsen (1993) have been widely recognized and cited. Authors who have published several publications are rare. Fourteen journals have a publishing rate over 1%. Only nine publications about HIT were identified.* Discussions.* China's usability research started relatively late. There is a lack of organized research teams and dedicated usability journals. High-impact theoretical studies are scarce. On the application side, no original and systematic research frameworks have been developed. The understanding and definition of usability is not well synchronized with international norms. Besides, usability research in HIT is rare.* Conclusions.* More human and material resources need to be invested in China's usability research, particularly in HIT.

## 1. Introduction

Usability is essential for the effective, efficient, and safe design, use, and learning of information technology. Research and application of usability have received significant attention by scientists, designers, and industry professionals in Western countries where there are active study populations, comprehensive theories, methods, practices, practical results, and mature industrial and professional organizations. In the field of health information technology (HIT), usability research has been identified as an important cognitive challenge for the adoption and meaningful use of HIT by the Office of National Coordinator for Health Information Technology (ONC), which is part of the U.S. Department of Health and Human Services (DHHS), and has become an active area for research, design, and practice in HIT [1–8]. Methods of usability evaluation have been demonstrated to improve the design and utilization of clinical information systems [9–11]. Usability, under the name of “safety enhanced design,” has become a requirement of Stage 2 meaningful use requirement for electronic health records (EHR) in the United States [12]. In China, with 30 years of rapid economic development, China's investment in social development and scientific research has been increasing dramatically. Given this context, one question we would like to answer is as follows. What is the current status of usability research in China? In this paper, through systematically reviewing the literature published in China and applying a combined qualitative and quantitative approach, we present the current status and existing problems of usability research and practice in China's information technology field.

## 2. Materials and Methods

### 2.1. Literature Search Strategy

#### 2.1.1. Chinese Publication Search Strategy

Usability is a broad and interdisciplinary field with inconsistent terminologies. This problem is compounded when translating these terms into Chinese because the words often have multiple meanings. After carefully reviewing the literature in August 2012, we decided to conduct a literature review using the Chinese equivalents of the following search terms: “usefulness,” “usability,” “user experience,” “user satisfaction,” and “user-centered design.” We used the advance search functions of following three mainstream databases in China: China National Knowledge Infrastructure (cnki.net), China Science and Technology Periodical Database (cqvip.com), and Wanfang Electronic Journal Database (wanfangdata.com.cn). We only searched the “title” and “keyword” fields in the literature published from the time period between 1980 and 2012.

#### 2.1.2. English Publication Search Strategy

Although our research was to review the literature originating from China using Chinese academic databases, we used the English language database, EBSCO, because it had the advanced search function that allowed us to select papers that originated from China. We searched using the advanced functionality provided by EBSCO, in the fields of “title” and “abstract” with any of the search terms “usability,” “user experience,” “user-centered design,” “user satisfaction,” “customer satisfaction,” “user interface,” “UCD,” and “China” and limited the results to those originating in China.

### 2.2. Literature Inclusion and Exclusion Criteria

#### 2.2.1. Inclusion Criteria

Inclusion criteria include (1) screening the published literature on usability in the field of HIT in China.

#### 2.2.2. Exclusion Criteria

Exclusion criteria include (1) excluding research literature about usability in other fields; (2) for duplicate entries, excluding those without complete information; and (3) excluding those without full text.

### 2.3. Data Extraction and Statistical Processing

**Figure 1:**
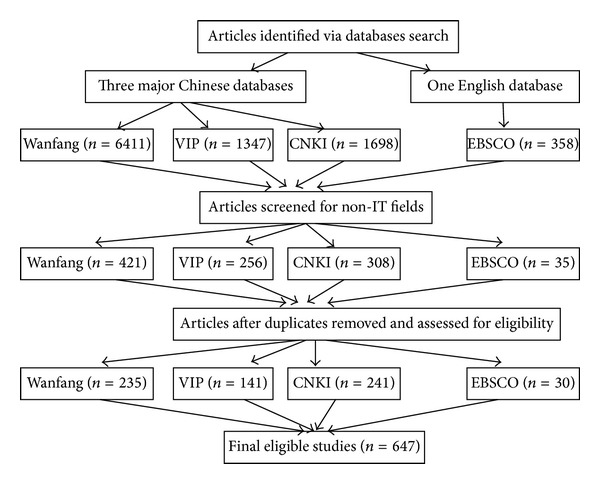
The PRISMA (preferred reporting items for systematic reviews and meta-analyses) flow chart.

The information summary sheet was designed to extract data from the selected literature. Information extracted included author, article title, year, journals, subcategory types for theoretical research, subcategory type for empirical studies, definition of usability, evaluation objects in empirical study, subcategory type of evaluation research in network application, and evaluation method used in empirical study. See Figure 1 for the PRISMA (Preferred Reporting Items for Systematic Reviews and Meta-Analyses) flow chart.

## 3. Results

### 3.1. Literature Search Results

Through searching of the literature for thirty-two years in the three databases and EBSCO, we obtained a total of 9814 publications. After applying our inclusion and exclusion criteria, 617 Chinese and 30 English publications were included in our analysis and accounted for 6.6% of the total retrieved literature. Reasons for the high exclusion rate are as follows.

1. Repeated retrievals: it is common that the same article may be retrieved in all three databases.
2. Study fields involved in the retrievals are unrelated. For example, topics on architecture, transportation, and so on rather than “information technology” were excluded.
3. Limitation of search function by certain database: for instance, the Wanfang database returned 6411 items, far more than 1347 and 1698 items from VIP and CNKI databases. The reason is that Wanfang is unique in the way that it does not support whole word search; rather, it will return all results containing each composite word of a complete word. In Chinese usability is composed of three “words.”
4. Excluded nontechnical items: for example, some items retrieved were advertisements released by companies for new products or news published on nontechnical magazines. The inclusion and exclusion process and the results are listed in Table 1.

### 3.2. General Characteristics of the Literature

#### 3.2.1. Year Distribution of the Literature

**Figure 2:**
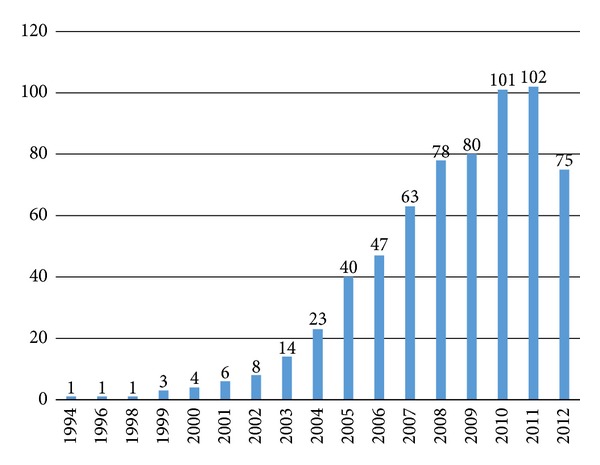
Distribution of the literature publications by year. Note: the abscissa represents the publication year and the ordinate represents the number of publications.

Publication dates of the 647 publications span from 1994 to 2012. The distribution across the years is shown in Figure 2.

#### 3.2.2. Type Distribution of the Literature

In the 647 items incorporated into our study, 564 are periodical publications and account for 87.2% of the total items, 52 are conference proceedings and account for 8.0%, 30 are theses/dissertations and account for 4.6%, and one was a book chapter accounting for 0.2%.

#### 3.2.3. Author Distribution of the Literature

Among the 1190 authors (including the second and the third coauthors) that contributed to the selected research publications, the following 10 authors listed in Table 2 published the most publications.

#### 3.2.4. Journal Distribution of Published Literature

Journals or conference proceedings in the 647 publications (top 14 with the highest quantity) are listed in Table 3.

#### 3.2.5. Distribution of Keywords Involved in the Literature

There are a total of 2229 keywords in the 647 publications. The keywords with the top 8 occurrence frequencies are listed in Table 4.

### 3.3. Results of Usability Research in China's Information Technology Field

#### 3.3.1. Types of Domestic Usability Research in Information Technology Field

Usability research in China may be roughly divided into two categories. The first category is the study about usability theories; there are a total of 395 publications accounting for 61% of the total retrieved publications. The other category includes empirical studies of usability, of which qualitative research methods were primarily utilized. The theoretical study mainly includes the following three aspects in contents: (a) usability history of development, influencing factors and problems encountered in usability (in different branch fields); (b) usability evaluation methods (in different branch fields); and (c) usability design principles and design concepts (in different branch fields).

Empirical studies related to usability accounted for 39% (i.e., 252) of the retrieved publications. Those publications related to the integration of usability during software or technology development account for 17% (i.e., 42) of the total retrieved publications. Most studies (83% or 210 total) focused on usability evaluations in specific target areas through the selected evaluation methods. The evaluation methods mostly applied are the combination of qualitative and quantitative studies.

**Figure 3:**
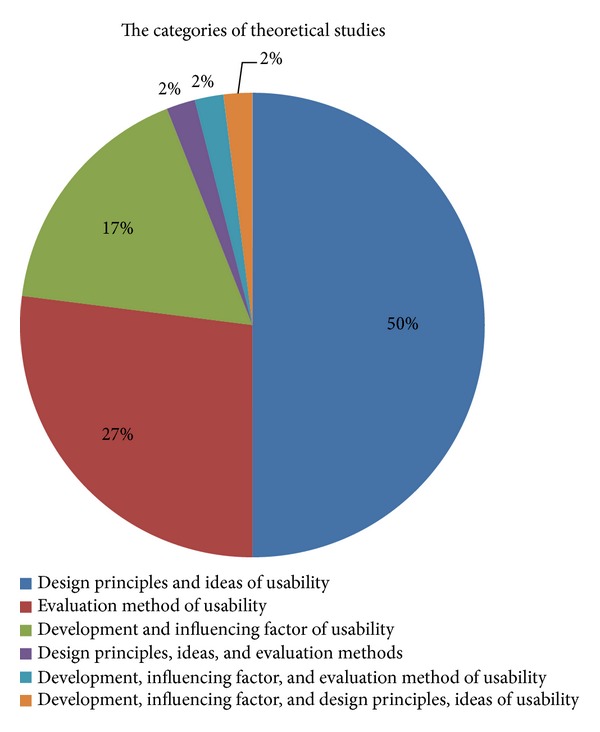
Classification of theoretical studies.

**Figure 4:**
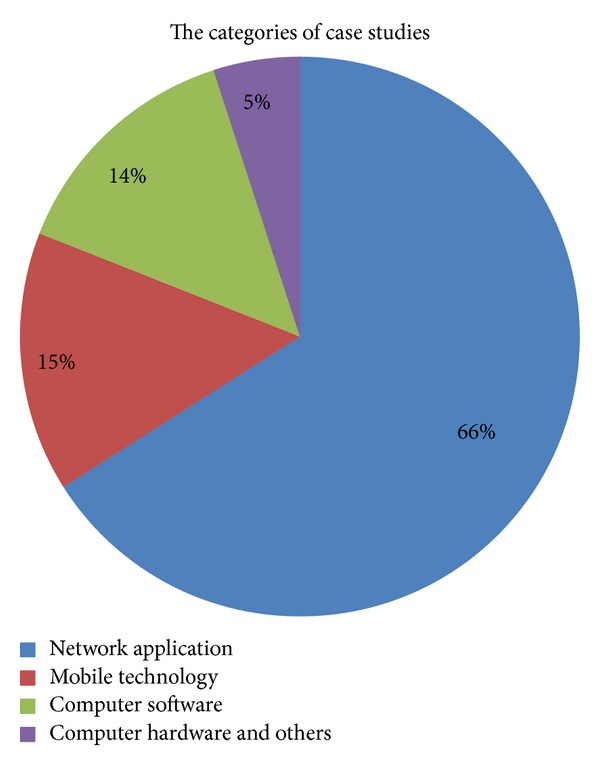
Classification of empirical case studies. Note: study targets in network application mainly include websites and network services; mobile study targets mainly include the interface design and applications of mobile phones and other mobile terminals.

The categorization of theoretical studies is shown in Figure 3. Classification of empirical studies is shown in Figure 4.

#### 3.3.2. Understanding of Chinese Researchers on the Concept of Usability

Based on the retrieved publications, the usability concepts in Table 5 are more commonly recognized by Chinese researchers.

Specific definitions are as follows.

- In the China national standard GB/T16260-2006 “Software engineering products quality,” usability is defined as “the ability of a software product to be understood, studied, used and as well as [sic] the ability to attract users in a particular use environment.”
- In the usability definition given by Nielsen in 1993 [13], usability includes 5 aspects, which are learnability, efficiency, memorability, errors, and satisfaction, respectively.
- In the ISO 9241-11:1998 [14] “Ergonomic requirements for office work with visual display terminals (VDTs),” usability is defined as “extent to which a product can be used by specified users to achieve specified goals with effectiveness, efficiency and satisfaction in a specified context of use.”
- In the ISO/IEC 9126-1:2001 [15] “Software products evaluation-quality properties and operation directions,” usability is defined as “the capability of the software product to be understood, learned and liked by the user, when used under specified conditions.”
- In recent years, with the advancement of usability research, new terminology emerges constantly. In the 647 publications, the “user experience, user satisfaction, and user-centered design” are also mentioned; among them, “user experience” is mentioned 118 times, “user satisfaction” 25 times, and “user-centered design” 12 times.

#### 3.3.3. Specific Types of Network Study Targets in Empirical Studies for Usability Evaluation

**Figure 5:**
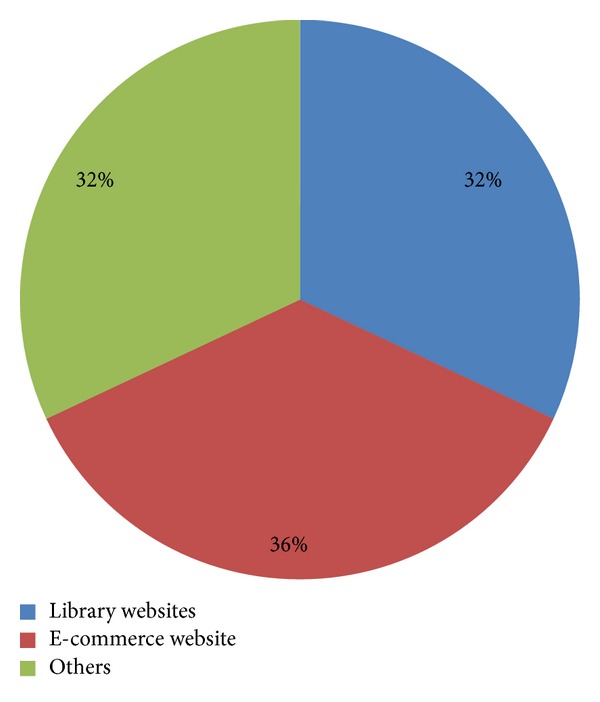
Distribution of study targets in web application study. Note: other targets mainly include other types of websites (government websites, various major portals, etc.), search engines, and network services.

Through analyzing the retrieved publications, we found that the Chinese empirical studies on usability focused mainly on website application (138 publications; see Figure 5 for the breakdown of website types).

Regarding the trend of evaluation objects, we can see from Table 6 that usability evaluation studies in web application still have a dominant position in recent years. However, usability evaluation studies on mobile Internet (including mobile phones) and applications did not increase as would be expected with the current rapid growth of mobile technology.

#### 3.3.4. Types of Study Methods in Empirical Studies for Usability Evaluation

Through analyzing the retrieved publications, we found that the usability evaluation methods used mainly include questionnaires, usability tests, heuristic evaluation, usability guidelines (such as MUG: Microsoft Usability Guideline), statistical analyses through system logs, cognitive walkthrough, behavior analyses, observation and interviews, eye movement analyses, distance of information-state transition (DIT), and other methods. The specific applications of these methods in the empirical studies are listed in Table 7.

In usability evaluation research, some studies may use two or more evaluation methods to obtain a greater understanding of the system's usability.  Table 8 provides a breakdown of the number of research methods used in each article.

#### 3.3.5. Studies in the Field of Healthcare Information Technologies

Of special note is that among the 647 publications only nine are about usability as it relates to HIT (specific information about the literatures is listed in Table 10).

## 4. Discussion

1. From our analyses of the publication dates and publication quantities, it is evident that China's literature on usability research in the information technology field began in 1994. The quantity of publications has been increasing year by year, starting with one publication identified in 1994 and culminating in a total of 102 publications in 2011. Publications released from 2010 to August 2012 account for 43% of the total publications. Our data show that usability research in China's information technology field started relatively late and the history is not long, but it is attracting more attention.
2. From the data on the authors and the publishing journals, we conclude that the targets of usability research in China's information technology field are relatively scattered and no coordinated usability efforts, such as research institutes or centers, have been established.
3. The most prolific author with the largest number of publications has only published 12 publications, and the top 10 authors with the most publications account for 11% (including the coauthors) of the total publications printed. The fields of the main publications in which the publications are produced are relatively simple and centralized. The top 11 journals that publish the most literature are mostly library information journals. The publication quantity distribution shows that the top 14 journals published 150 publications about usability research, accounting for 23.3% of the total publications. This shows that, in the information technology usability research field, articles are published across a diverse spectrum of journals and there is no journal with a major focus on usability. Of course, these journals are respectively included in the “China Journal Citation Report” by the China Science and Technology Information Institute and the “Chinese core journals” of the Peking University, which are believed to have a good reputation. In the United States, there are many pioneers and authorities in the field of usability, such as Jakob Nielsen, who have produced many publications, but China has yet to have such established and prolific experts on these topics. What is more, besides specific peer reviewed journals for usability, such as “Journal of Usability Studies”, there are more than 50 types of journals/magazines which are directly associated with usability.
4. The data on study types show that theoretical studies on usability account for 61% of the total publications, including 27% about usability evaluation methods. Through analyzing the retrieved literature, we found that theoretical studies typically combine usability with specific branch fields (such as study of usability in web application) in terms of usability influencing factors, usability problems, usability design principles, and usability evaluation methods. However, the theoretical studies typically do not have significant original proposals or innovative methods and frameworks. At present, major advancements in theoretical and methodological studies are lacking in China.
5. From the data on the understanding on usability concepts, we found that the Nielsen [13] and ISO 9241-11:1998 [14] definitions account for 66.1% of the publications, showing that these two definitions are widely recognized by Chinese researchers. However, the above two definitions were proposed in earlier years. Based on the newest usability definition provided in ISO 25010:2011, “System and software quality model”, usability is not only a property about product quality but also a property about quality in use of the project (comprising effectiveness, efficiency, and satisfaction) [16]. Yet, this newest definition is not mentioned in any current Chinese usability literature, so, to a certain extent, this may demonstrate that Chinese researchers are falling behind on international usability research. A new definition of usability was just proposed last year by Zhang and Walji [5], with the intent to unify all the variations of usability definitions, concepts, and applications under a single theoretical framework.
6. From the data on study targets, we can see that most Chinese usability studies are combined with evaluation studies and the evaluation objects are mainly focused on Internet applications. In evaluation studies, 66% are usability studies in Internet applications; this is partially because usability studies on Internet started relatively early in western countries. Many usability experts in web applications have proposed various website usability evaluation methods and practice guidelines. For example, in the US, Story argues that a website developer should follow 10 usability principles when the site is designed [17]. The Northwest Alliance for Computational Science and Engineering (NACES) formulated common website usability guidelines for website design, webpage design, and navigation help [18]. Borges et al. from University of Puerto Rico also proposed 16 usability principles for web design and proved the effectiveness of these principles by experiments [19]. Nielsen, a pioneer in usability research, conducted many important studies on usability of websites, addressing theories, methods, practice, and other aspects of usability [13, 20, 21].
7. Meanwhile, with the development of digital multimedia technology and wireless network technology, evaluation objects in usability evaluation studies are also changing gradually.
8. From the data on usability methods we can see that evaluation methods are divided into two categories: usability testing and questionnaires. They account for 58.2% of all evaluation methods. In most cases, a single method is applied in usability evaluation and this accounts for 63.8% of the evaluation methods. The data on combination of different evaluation methods are listed in Table 9. Obviously, usability testing and questionnaires, as two prominent methods, appear to play an important role in usability research. Although each evaluation method has its own use conditions, combining multiple methods may evaluate usability from a more comprehensive perspective. Combination of multiple methods will likely become a development trend for use of evaluation methods in the future.
9. Further analysis of the data on distribution of study targets shows that Chinese usability studies are mostly concentrated on web information technology and usability studies in the healthcare information technology field are quite limited. Among the 647 publications, only nine are about this field (specific information about the literatures is listed in Table 10). Comparatively, as of 25 November 2012, the biomedical database PubMed returned about 942 publications (search from titles) and 4861 publications (search from abstracts) preliminary search results using the similar combination of query terms: “usability or user experience/s or user centered design or user satisfaction.” Compared with the rapid development of the HIT industry in China, usability research in HIT in China is very underdeveloped. Many studies have shown that usability improvement of HIT could effectively reduce medical errors [20], thus improving patient quality. Obviously, it is both important and urgent to carry out usability research in China's HIT field.

## 5. Study Limitations

This paper studies the current status and characteristics of usability research in China's information technology field by using the systematic review and a quantitative literature analysis. Limitations of the study are listed as follows. (1) Usability research is an interdisciplinary field and researchers in different disciplines often use different terminologies. To minimize any effects of overrepresentation, we used many different keywords such as “usability,” “user satisfaction,” “user experience,” and “user-centered design” to query the related literature. (2) Three major databases are used in our study and we searched via the field of keywords. However, keywords in Chinese publications do not have corresponding vocabulary similar to Mesh, and all keywords in Chinese publications are manually added by the author. We had to assume that if the authors had considered the subject of the article to be mainly about usability, they would have used one of the above related usability terms in the keywords, especially given the frequent references to Nielsen [13] and ISO 9241-11:1998 [14]. (3) This paper is limited to the information technology field, but typical publications may not explicitly use the keywords such as information technology in the titles or keywords; thus, we cannot enter “information technology” in the search query. We could only manually screen publications about information technology after all usability related publications are retrieved; this process needs more manual efforts. (4) Finally, this study focuses on the academic literature only; thus, the results obtained here are not inclusive. Furthermore, as usability is also an application intensive discipline, it is possible that usability related efforts are more active in industrial society than in academic domain reflected from this research.

## Figures and Tables

**Table 1:** Inclusion and exclusion process.

Databases	Wanfang database	VIP	CNKI	EBSCO	Total
Retrievals from database	6411	1347	1698	358	9814
After exclusion of repeated and irrelevant	235	141	241	30	647
screening rate	3.7%	10.5%	14.2%	8.8%	6.6%

**Table 2:** General author information.

Author	Institutional affiliation	Number of publications	Percentage
Liu, Zhenjie	EU Usability Chinese Center, Dalian Maritime University	12	1.9%
Qiu, Minghui	Consulting and Management Department, Sun Yat-sen University	8	1.2%
Ge, Liezhong	Psychological Department, Zhejiang University of Technology	8	1.2%
Zhang, Kan	Psychological Institute, Chinese Academy of Sciences	7	1.1%
Rob Law	Hong Kong Institute of Technologies	7	1.1%
Zhang, Liping	EU Usability Chinese Center, Dalian Maritime University	6	0.9%
He, Guihe	School of Economics and Management, Jingchu University of Technology	6	0.9%
Sun, Qingzhen	Zhengzhou Institute of Aeronautical Industry Management	6	0.9%
Huang, Xiaobin	Consulting and Management Department, Sun Yat-sen University	6	0.9%
Ren, Zhongbin	Institute of Surveying and Mapping, Information Engineering University	6	0.9%

Note: quantities of published literature of other authors were all less than 0.5% and are not listed here.

**Table 3:** List of journals and conference proceedings.

Journal or conference name	Number of publications	Percentage	Core journals∗
Ergonomics	23	3.6%	Peking University core and Technology core
Information science	13	2.0%	Peking University core
Library and information service	13	2.0%	Peking University core
Packaging engineering	12	1.9%	Peking University core
Modern library and information technology	12	1.9%	Peking University core
Art and design	12	1.9%	
Library studies	10	1.5%	Peking University core
Programmer	9	1.4%	
Intelligence theory and practice	9	1.4%	Peking University core
Computer engineering and applications	8	1.2%	Peking University core and Technology core
Market modernization	8	1.2%	Peking University core
Computer engineering and design	7	1.1%	Peking University core and Technology core
Computer science	7	1.1%	Peking University core and Technology core
Modern information	7	1.1%	Peking University core

Note: the publication quantity of other journals or conference proceedings is less than 1% and is not listed here. *aka “Peking University core journal” refers to the classification by Peking University Library on Chinese academic journals, published every 3-4 years and currently widely recognized by the Chinese academia. Publications in core journals are viewed with relative high academic levels and this is an important part of the academic evaluation system in China.

**Table 4:** Information about keywords in literatures.

Keywords	Publication usage frequency	Percentage
Usefulness	140	6.3%
User experience	84	3.8%
User satisfaction	28	1.3%
High usability	26	1.2%
Usability assessment	23	1.0%
Usability test	23	1.0%
Usability	21	0.9%
Website	21	0.9%

Note: occurring percentage of other keywords less than 0.9% is not listed here.

**Table 5:** List of usability concepts.

Usability definition	Mentioning rate of the definitions	Percentage
Definition given in ISO9241-11 “Ergonomic requirements for office work with visual display terminals (VDTs)”	151	36.8%
Definition given by Nielsen in 1993	120	29.3%
Other definitions (definitions in various branch fields by combining with specific study contents in the field)	120	29.3%
Definition given in ISO9126-1:2000 “Software Product Evaluation: Quality Characteristics and Guidelines for their Use-standard”	14	3.4%
Definition given in China national standard GB/T 162602006 “Software engineering products quality”	5	1.2%

Total	410	100%

Note: among the 647 articles, 282 did not provide definite usability definition. Among the articles providing usability definitions, an article may provide more than one definition.

**Table 6:** Distribution of evaluation objects by years.

Year	Classification of evaluation targets in usability evaluation study				Total (literature quantity)
	Web application	Mobile technology	Computer applications	Computer hardware	
1996	0	0	1	0	1

2002	0	0	2	0	2
2003	2	0	1	0	3
2004	3	0	2	2	7
2005	4	3	1	2	10
2006	15	3	0	0	18
2007	19	3	4	3	29
2008	23	6	2	1	32
2009	14	4	4	0	22
2010	23	3	6	1	33
2011	21	5	3	1	30
2012	14	4	4	1	23

Total (literature quantity)	138	31	30	11	210

**Table 7:** List of usability evaluation methods.

Usability evaluation method	Application times in study	Percentage
Questionnaires	90	30.8%
User/researcher usability test	80	27.4%
Following existing guidelines (such as MUG)	61	20.9%
Eye movement analysis and DIT theory and other methods	33	11.3%
Observation and interviews	12	4.1%
Heuristic evaluation	11	3.8%
Statistical analysis through system log files	4	1.4%
Cognitive walkthrough	1	0.3%

Total	292	100%

Note: multiple evaluation methods may be used in the same study.

**Table 8:** Combination uses of evaluation methods.

Use of evaluation methods	Quantity of literatures involved	Percentage
Single evaluation method	106	58.2%
Two evaluation methods	66	36.2%
Three or more evaluation methods	10	5.6%

Total	182	100%

**Table 9:** Specific combinations between evaluation methods.

Combination use of evaluation methods	Number of publications involved	Percentage in literature about evaluation study
Questionnaires and following existing guidelines (such as MUG)	29	13.8%
Questionnaires and user/researcher usability testing	15	7.1%
User/researcher usability testing and observation/interviews	5	2.4%
User/researcher usability testing and following existing guidelines (such as MUG)	5	2.4%
User/researcher usability testing and heuristic evaluation	4	1.9%
Questionnaires and user/researcher usability testing and observation/interviews	4	1.9%
User/researcher usability testing and eye movement analysis and DIT theory	3	1.4%
Questionnaires and heuristic evaluation	2	1.0%
Heuristic evaluation and following existing guidelines (such as MUG)	1	0.5%
User/researcher usability testing and heuristic evaluation and cognitive walkthrough	1	0.5%
Questionnaires and user/researcher usability testing and guidelines (such as MUG)	1	0.5%
Questionnaires and user/researcher usability testing and heuristic evaluation	1	0.5%
Questionnaires and user/researcher usability testing and following existing guidelines (such as MUG) and statistical analysis through system log files.	1	0.5%
Questionnaires and heuristic evaluation and observation/interviews	1	0.5%
Following existing guidelines (such as MUG) and eye movement analysis and DIT theory and other methods	1	0.5%
Following existing guidelines (such as MUG) and statistical analysis through system log files.	1	0.5%
User/research usability testing and following existing guidelines (such as MUG) and eye movement analysis and DIT theory	1	0.5%

Total	76	36.2%

**Table 10:** Summary about literatures involving usability study in the health information technology.

Literature title	Year	Type	Contents studied
Telehealth for older patients: the Hong Kong experience	2002	Empirical study	Evaluation study about usability
Maintaining high usability of database, ensuring stable operation of hospital information systems	2005	Theoretical study	Usability design principles
Usability design study on human-machine interface of medical equipment	2007	Empirical study	Usability-oriented system software or technology development
Design on high usability of hospital information systems	2008	Empirical study	Usability-oriented system software or technology development
Study on user experience testing of China Disease Prevention and Control Center website in 2009	2010	Empirical study	Evaluation study about usability
Practice and improvement of clinic HIS high usability programs	2011	Theoretical study	Usability development and influence factors
Achieving high reliability and high usability of regional health information system database through ESX4	2012	Empirical study	Usability-oriented system software or technology development
Using recommendation to support adaptive clinical pathways	2012	Empirical study	Evaluation study about usability
A mobile nursing information system based on human-computer interaction design for improving quality of nursing	2012	Empirical study	Usability-oriented system software or technology development
